# Plasma Urotensin II levels in children and adolescents with chronic kidney disease: a single-centre study

**DOI:** 10.1186/s12882-017-0530-9

**Published:** 2017-03-31

**Authors:** Anastasia Garoufi, Styliani Drapanioti, Antonios Marmarinos, Varvara Askiti, Andromachi J. Mitsioni, Maria Mila, Georgia Grigoriadou, Dimitrios Georgakopoulos, Constantinos J. Stefanidis, Dimitrios Gourgiotis

**Affiliations:** 1 Second Department of Pediatrics, Medical School, National and Kapodistrian University of Athens,“P. & A. Kyriakou” Childrens’ Hospital, Thivon & Levadias str, 11527 Athens, Greece; 2 Laboratory of Clinical Biochemistry - Molecular Diagnostic, Medical School, National and Kapodistrian University of Athens, “P. & A. Kyriakou” Childrens’ Hospital, Thivon & Levadias str, 11527 Athens, Greece; 3 Department of Nephrology, “P. & A. Kyriakou” Childrens’ Hospital, Thivon & Levadias str, 11527 Athens, Greece; 4 Thivon & Levadias str, 11527 Athens, Greece

**Keywords:** Renal transplantation, End-stage kidney disease, Hemodialysis, Renal failure, Acidosis

## Abstract

**Background:**

Increased plasma Urotensin II (UII) levels have been found in adults with renal diseases. Studies in children are scarce. The objective of the study is to estimate plasma UII levels in subjects with chronic kidney disease (CKD) stages 3 to 5 and renal transplant recipients (RTR). In addition, the correlation of UII with anthropometric features and biochemical parameters was assessed.

**Methods:**

Fifty-four subjects, aged 3 to 20 years old, 23 with CKD, 13 with end-stage kidney disease (ESKD) undergoing hemodialysis (HD) and 18 RTR were enrolled. A detailed clinical evaluation was performed. Biochemical parameters of renal and liver function were measured. Plasma UII levels were measured in all patients and in 117 healthy controls, using a high sensitive enzyme immunoassay (EIA) kit. All data were analyzed using STATA™ (Version 10.1).

**Results:**

Median UII and mean log-transformed UII levels were significantly higher in CKD and RTR patients compared to healthy subjects (*p* < 0.001). HD patients had higher but not statistically significant UII and log-UII levels than controls. UII levels increased significantly at the end of the HD session and were higher than controls and in line to those of other patients. The geometric scores of UII in HD (before dialysis), CKD and RTR patients increased respectively by 42, 136 and 164% in comparison with controls. Metabolic acidosis was associated with statistical significant change in log-UII levels (*p* = 0.001). Patients with metabolic acidosis had an increase in UII concentration by 76% compared to those without acidosis.

**Conclusions:**

Children and adolescents with CKD, particularly those who are not on HD and RTR, have significantly higher levels of UII than healthy subjects. UII levels increase significantly at the end of the HD session. The presence of metabolic acidosis affects significantly plasma UII levels.

## Background

Urotensin II (UII) is the most potent vasoconstrictor peptide in humans. It has also been reported to have a vasodilatory effect on small arteries of rats as well as on resistance arteries of humans [1–3]. It is synthesized mainly in the kidney, but also in non-renal tissues such as the heart, liver, pancreas and adrenal glands [4]. The high levels of plasma UII in surgically anephric patients is a strong indication of extrarenal production [5].

Increased plasma UII levels have been found in many pathological conditions such as hypertension, cirrhosis, congestive heart failure and renal failure, but the role of UII in human diseases, including renal disease, is still controversial [6–14]. Initial evidence suggested that UII contributes to the development of cardiovascular diseases as well as renal dysfunction, however in the last decade many studies have shown a possible cardioprotective role of high UII levels in patients with ischemic heart disease and chronic kidney disease (CKD) [11, 15, 16]. Furthermore, low UII levels have been associated with cardiovascular events and death in adults with CKD [17, 18].

There is limited information on UII levels in children. There are few reports on UII levels in children and adolescents with pulmonary hypertension, portal hypertension, congenital heart disease and nephrotic syndrome [19–23].

To our knowledge this is the first study of plasma UII levels in children and adolescents with CKD of stages 3–5, ESKD on dialysis and renal transplantation.

The aim of the present study was to evaluate prospectively plasma UII concentration in the above pediatric population; its association with management options (dialysis or no dialysis, renal transplantation) and its correlation with anthropometric features, hemodynamic and biochemical parameters.

## Methods

### Study population

In this single - centre prospective study, fifty-four Caucasian children and adolescents (33 boys, 21 girls), aged 3 to 20 years old, twenty-three with CKD stages 3–5, thirteen with ESKD who were on regular dialysis treatment and eighteen RTR, were enrolled. The mean age had no statistically significant difference between the three groups (*p* 0.424). The underlying causes of chronic kidney disease include: Hypodysplasia ± reflux nephropathy (*n* = 23), obstructive uropathy (*n* = 6), glomerulonephritis (*n* = 6), congenital kidney and urinary tract anomalies (CAKUT, *n* = 5), cystic kidney disease (*n* = 3), congenital nephrotic syndrome (*n* = 3), hereditary nephropathy (*n* = 3), hemolytic uremic syndrome (HUS, *n* = 2), miscellaneous(*n* = 1), unknown (*n* = 2). One hundred and seventeen age-matched (*p* = 0.242) healthy children and adolescents comprised the control group. Patients and controls were recruited from the Department of Nephrology and from the 2nd Department of Pediatrics of Athens University of the “P. & A. Kyriakou” Children’s’ Hospital respectively. The study was conducted from May 2012 to February 2014 and was approved by the Hospital’s Ethics Committee prior to its initiation. All participants’ parents or legal guardians were required to complete a consent form. Parents of only one child with CKD refused to participate in the study. The following exclusive criteria were mandatory for the patients group: children below 3 years of age, CKD stages 1 and 2, duration of dialysis and transplantation less than 2 and 6 months respectively and those with co-occurrence of hepatic, pulmonary or congenital heart disease.

### Definition and classification of CKD

For the definition of CKD we used the criteria recommended by Kidney Disease Quality Outcome Initiative (K/DOQI) [24]. CKD staging was based on the estimated Glomerular Filtration Rate (eGFR) according to the KDOQI CKD classification. The eGFR was calculated in ml/min/1.73 m^2^ according to Schwartz formula [25]. Graft function was also classified to CKD 1–5 stages.

### Anthropometric features, clinical evaluation and medical data

In all participants, anthropometric features, personal and family history were evaluated; and a detailed physical examination was carried out. The body weight (BW) was measured to the nearest 0.1 kg using an electronic scale (SECA) and the height (Ht) to the nearest 0.1 cm by a wall stadiometer (Hyssna). BSA was calculated in m^2^.

The “dry weight” was also estimated in patients undergoing dialysis with the use of a body composition monitor. Body mass index (BMI) was calculated by the equation: BW (kg) per Ht (m^2^). Participants were classified as normal BMI, overweight (OW) and obese (OB) using the International growth charts [26]. Moreover, standard deviation scores (SDS) for BW, Ht and BMI were calculated using a standardized age- and sex- specific calculator. Blood pressure (BP) was measured by an electronic automated oscillometric device Dynamap with a suitable cuff size (Critikon) for the child’s arm circumference. Three BP measurements (with one minute interval) were taken in a sitting position after a 5 min rest. The average of these measurements was used in the analysis. The systolic BP (SBP) and diastolic BP (DBP) values were classified using chart percentiles for age, sex and height and hypertension was defined as BP equal or above the 95th percentile [27]. SDS for SBP and DBP were also calculated by a standardized calculator based on age, sex and height. A full cardiological evaluation was performed in all patients. Left ventricular mass (LVM) and left ventricular mass index (LVMI) were estimated.

Twelve out of 13 patients underwent conventional hemodialysis three times a week and one patient four times a week, approximately 4.5 h per session. The dialysis machines that were used were Gambro AK200S and Nikisso model dbb 05. Dialysers were chosen according to body surface area (BSA). In these subjects, BW, BMI and BP were re-evaluated after the completion of dialysis.

The mean duration of hemodialysis (HD) was 1.2 (SD 0.9) years. The mean duration of transplantation was 5.6 (SD 2.2) years. All RTR patients were on immunosuppressive drug treatment with prednisolone, mycophenolate and cyclosporine or tacrolimus.

### Laboratory tests

Venous blood samples were collected between 8–9 am, after an overnight fast in all participants. All children were infection free for at least 10 days before the examination.

Biochemical parameters such as urea, creatinine, total protein, albumin, uric acid, electrolytes, transaminases and γGT were measured in serum using standard laboratory methods. Cystatin C was measured using an automated particle-enhanced nephelometric immunoassay (PENIA) on a Siemens Behring Nephelometer BN II system. Venous blood gases and a full blood count were obtained.

We defined anemia as levels of hemoglobin below the lowest limit of normal for age and sex. Metabolic acidosis was defined as pH <7.35 and base < 22 mmol/L.

For the determination of UII, whole venous blood in EDTA tubes was collected; it was immediately placed in ice and then centrifuged at 1600 g for 10 min at 4 °C. The supernatant was stored in aliquots of 200 μl at -70 °C until the time of analysis. Plasma UII levels were measured by a high sensitive enzyme immunoassay (EIA) kit (Phoenix Pharmaceuticals, Inc. 330 Beach Road, Burlinghame, CA 94010, USA) and expressed in ng/ml. The intra-assay and the inter-assay variation CV% were <5% and <14% respectively. The range was 0–100 ng/ml, the linear range 0.06–1.48 ng/ml, and the detection limit 0.06 ng/ml.

In patients undergoing hemodialysis the markers of renal function and plasma UII concentration were evaluated twice, before the initiation and after the completion of midweek dialysis session.

### Statistical analysis

The Shapiro-Wilk test was performed to test for normal distribution of continuous variables. The results are given as mean (+/- SD) or as median and interquartile range (IQR) according to normality of relative frequencies. All qualitative variables are presented as absolute or relative frequencies.

The Student’s t-test or its non-parametric equivalent Mann-Whitney U test was used to compare continuous variables between the groups under study. The Fisher’s exact test was employed for comparison of categorical variables. One way analysis of variance (ANOVA) or Kruskal Wallis test was used for comparison of parametric and non-parametric variables between groups, respectively. Sidak correction was used for multiple comparisons.

**Fig. 1:**
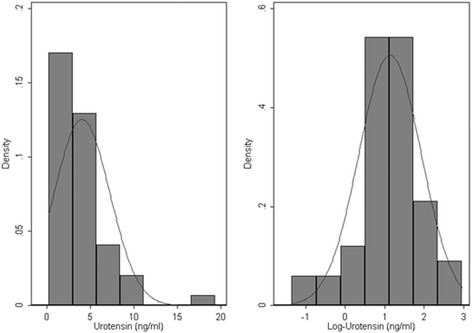
Distribution of plasma Urotensin II levels and log-transformed UII levels in patients

Urotensin II levels had to be log-transformed in order to achieve a normal distribution in patients (Fig. 1) and healthy controls (data not shown).

Log transformed UII values were used as the dependent variable for the linear regression analysis and in multiple regression models in order to investigate the relationship between UII and a series of factors. Since log-transformed UII was used all interpretations are presented as proportion change in geometric mean of UII values.

Study groups and each one of the variables such as age, disease duration, markers of renal function (e.g. eGFR, creatinine, cystatin C and electrolytes) and hemodynamic parameters (SBP, DBP, heart rate, LVM and LVMI), were used as independent variables. The non parametric Spearman correlation coefficient was applied in order to associate UII levels with several factors.

All tests were two-tailed and statistical significance was established at *p* = 5%. Data were analyzed using STATA™ (Version 10.1 MP, Stata Corporation, College Station, TX, 77845, USA).

## Results

The anthropometric features and clinical characteristics of the three groups of patients are shown in Table 1. Children undergoing HD had greater growth retardation and greater percentage of hypertension compared to other groups (Table 1). Moreover, Systolic and Diastolic BP as well as SDS of SBP and DBP were lower but the differences had no statistical significance (*p* = 0.119, *p* = 0.148, *p* = 0.145 and *p* = 0.131 respectively). A significant decrease in body weight (1.19 ± 0.85 kg) after the completion of HD was established (*p* = 0.001).

**Table 1:** Clinical and anthropometric characteristics of patients

	CKD (*n* = 23)	HD (*n* = 14)	RTR (*n* = 17)	*P* value*
Gender (Males %)	74	54	50	0.284
Age (years)	11.5 (4.8)	10.3 (3.6)	12.3 (3.8)	0.424
eGFR (ml/min/1.73 m^2^)	31.0 [22, 47]^a^	6.0 [6, 7]^b^	58.5 [47, 63]^c^	<0.001
Disease duration (yrs)	8.9 (4.8)	7.6 (4.4)	9.8(3.2)	0.391
SDS-Weight	-0.44 [-1.23, 0.62]^a^	-1.84 [-3.41, -0.86]^b^	0.41 [-0.37, 0.75]^a^	0.001
SDS-Height	-0.90 (1.01)^a^	-2.92 (1.60)^bc^	-1.98 (0.94)^c^	<0.001
BSA (m^2^)	1.24 (0.42)^a^	0.90 (0.23)^b^	1.27 (0.32)^ac^	0.012
BMI (kg/m^2^)	18.8 [16.9, 21.9]^ab^	15.9 [14.9, 18.4]^a^	21.7 [18.4, 26.7]^b^	0.004
SDS-BMI	0.41 [0.02, 1.2]^ab^	-0.70 [-1.29, 0.53]^a^	1.26 [0.61, 1.79]^b^	0.006
BMI (Increased %)	26.1	26.1	72.2	0.004
Fat (%)	19.7 [14.7, 23.9]	13.7 [12.6, 19.3]	20.2 [15.6, 24.5]	0.125
Lean Body Mass (kg)	34.2 [20.3, 41.8]^a^	19 [17.7, 24.2]^b^	35.8 [25.9, 43.9]^a^	0.004
SDS-dry Weight		-2.55 [1.97]		
BMI-dry weight (kg/m^2^)		14.9 [14.5,18.1]		
SDS-BMI (dry weight)		-0.85 [1.7]		
Systolic BP (mmHg)	103.5 (16.4)	111.9 (21.0)	113.6 (12.9)	0.128
SDS -Systolic BP	-0.002 (1.30)^a^	1.36 (2.08)^b^	1.14 (0.98)^b^	0.011
Diastolic BP (mmHg)	60.2 (11.7)^a^	70.9 (16.9)^ab^	71.1 (8.6)^b^	0.009
SDS- Diastolic BP	-0.02 (1.13)^a^	1.06 (1.32)^b^	0.96 (0.73)^b^	0.004
Increased BP (%)	17.4	61.5	16.7	0.012

*CKD* chronic Kidney Disease, *HD* Hemodialysis, *RTR* Renal Transplant Recipients, *eGFR* estimated Glomerular Filtration Rate, *BSA* Body Surface Area, *BMI* Body Mass Index, *SDS* Standard Deviation Scores, *BP* Blood Pressure, Increased BP: above the 95th percentile for gender, age and height. Superscript letters a, b, c denote values of different statistical significance

*Results are presented as mean (SD) or as median [IQR] according to data distribution. Tests employed are one way ANOVA and Kruskal – Wallis test, respectively. For qualitative data Fisher’s exact test was applied. Tests employed are paired t test or Mann–Whitney U test, respectively

Laboratory data for three study groups are shown in Table 2. Children on HD had significantly lower mean hemoglobin and hematocrit than CKD group. Moreover, anemia was more common in HD group, compared to other groups. In addition, serum chloride was lower in HD than other groups and serum potassium was significantly higher only compared to RTR group. Finally, serum albumin concentration was lower in HD children compared to CKD patients (Table 2).

**Table 2:** Laboratory data of the patient groups

	CKD (*n* = 23)	HD (*n* = 14)	RTR (*n* = 17)	*P* value*
Urea (mg/dl)	78 [49, 128]^a^	162 [135, 203]^b^	47.5 [37, 66]^c^	<0.001
Creatinine (mg/dl)	1.7 [1.3, 2.3]^a^	7.4 [6.8, 9]^b^	1 [0.8, 1.2]^c^	<0.001
Cystatin (mg/L)	1.85 [1.53, 3.25]^a^	6.54 [6.15, 6.96]^b^	1.42 [1.04, 1.67]^c^	<0.001
Uric acid (mg/dl)	7.1 (1.85)^a^	7.0 (1.36)^ab^	5.88 (1.34)^b^	0.010
Sodium (mEq/L)	138 [137, 140]	137 [136, 139]	138 [137, 139]	0.426
Potassium (mEq/L)	4.6 [4.4, 5]^ab^	5 [4.7, 5.5]^a^	4.4 [4.2, 4.7]^b^	0.011
Chloride (mEq/L)	103 [101, 104]^ac^	97.5 [91.5, 99]^b^	104 [102, 108]^c^	<0.001
Ca (mg/dl)	9.9 [9.7 , 10.2]	10.1 [9.7, 10.3]	10 [9.7, 10.3]	0.860
P (mg/dl)	4.4 [4.1, 4.9]	5 [4.5, 6.9]	4.1 [4.0, 4.3]	0.008
ALT (U/L)	22 [18, 28]	18 [17, 23]	17.5 [17, 19]	0.174
AST (U/L)	14 [12, 21]	16 [12, 24]	13.5 [11, 16]	0.274
γGT (U/L)	12 [10, 14]	12 [8, 48]	12 [10, 16]	0.951
Total Protein (g/dl)	7.39 (0.54)^a^	6.78 (0.47)^b^	6.72 (0.62)^b^	<0.001
Albumin (g/dl)	4.59 (0.23)^a^	4.28 (0.33)^b^	4.48 (0.30)^ab^	0.010
pH	7.35 (0.03)	7.37 (0.04)	7.35 (0.03)	0.134
HCO3 (mEq/L)	22.0 (2.57)	24.4 (4.10)	22.19 (3.67)	0.147
Acidosis (Yes %)	39.1	15.4	41.2	0.263
Hct (%)	37.4 (4.14)^a^	32.7 (4.62)^b^	35.6 (5.14)^ab^	0.018
Hb (g/dL)	12.5 (1.53)^a^	10.7 (1.40)^b^	11.7 (1.66)^ab^	0.008
Anemia (Yes %)	34.8	76.9	55.6	0.051

*CKD* chronic Kidney Disease, *RTR* Renal Transplant Recipients, *HD* Hemodialysis. *Results are presented as mean (SD) or as median [IQR] according to data distribution. Superscript letters a, b, c denote values of different statistical significance

**Fig. 2:**
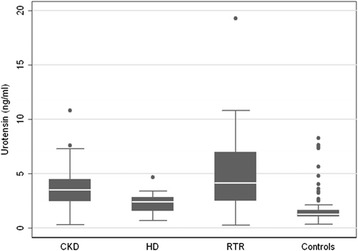
Box plot of UII levels by group. CKD: chronic Kidney Disease, HD: Hemodialysis, RTR: Renal Transplant Recipients

Median UII and mean log-transformed UII levels were significantly higher in CKD and RTR patients compared to healthy controls (*p* < 0.001 and *p* < 0.001). HD patients had also higher UII and log-UII levels than controls but there was not statistical significance (Fig. 2, Table 3). The increase of the mean of UII scores, by switching from controls to HD, CKD and RTR patients, was 42, 136 and 164% respectively. There was also an increase in the mean of UII scores of 86% by switching from HD to RTR patients (*p* < 0.05).

**Table 3:** Comparisons between patient groups and controls for UII and log-transformed UII values

	CKD (*n* = 23)	HD (*n* = 14)	RTR (*n* = 17)	Controls (*n* = 117)	*P* value*
Urotensin II	3.53 [2.49, 4.46]^a^	2.42 [1.60, 2.79]^ab^	4.16 [2.53, 6.96]^a^	1.28 [1.08, 1.63]^b^	<0.001
Log - Urotensin II	1.22 (0.71)^acd^	0.71 (0.53)^bd^	1.33 (0.95)^c^	0.36 (0.54)^b^	<0.001

Tests employed are one way ANOVA, and Kruskal – Wallis test, respectively. Different letters denote statistical significant difference between groups (*p* < 0.05)

*CKD* Chronic Kidney Disease, *HD* Hemodialysis, *RTR* Renal Transplant Recipients

*Results are presented as mean (SD) or as median [IQR] according to data distribution

Finally, in HD patients UII and log-transformed UII were increased significantly after the completion of dialysis [2.42 (1.6–2.79) vs 4.38 (2.17–7.62), *p* = 0.043 and 0.71 (SD 0.53) vs 1.45 (SD 0.96), *p* = 0.022 respectively) and there was no significant difference compared to other patient groups. Moreover, an increase of approximately 197% in the mean of U II scores is observed (*p* < 0.05) when switching from controls to HD patients measured at the end of their dialysis session. There was no correlation found between BW, Dry BW and their SDS with UII levels before and after the completion of dialysis. Furthermore, the increase of UII did not correlate with the decrease of body weight (*p* = 0.640), the improvement of renal function and the decrease of blood pressure.

There was no statistically significant difference in log-UII levels according to sex, age and body mass index in patients and controls.

UII levels in patients were negatively correlated with serum creatinine (r -0.37, *p* = 0.006) and cystatin C (r -0.44, *p* = 0.001). In addition, Spearman’s correlation coefficient was applied for the same pairs of factors per group. No statistical significant association was detected between any pair of factors (*p* > 0.05). For this reason, CKD and HD were merged, resulting in a significant negative correlation of UII with creatinine (r -0.46, *p* = 0.004) and cystatin C (r -0.41, *p* = 0.001) and a positive one with serum albumin levels (r 0.36, *p* = 0.032).

Linear regression analysis revealed that metabolic acidosis was associated with a statistical significant change in log-transformed UII levels (*p* = 0.010). More specifically, by switching from non-acidosis to acidosis a 76% increase in the mean of UII scores is observed. For a 0.1 units increase of pH and for a 1 mEq/L increase of HCO3, a decrease of approximately 50 and 7% respectively in the mean of UT II scores is expected (Table 4).

**Table 4:** Linear regression analysis of log-UT II adjusted for pH, HCO3 and metabolic acidosis by group*

Factor	Coefficient	S.D	*P*-value	95% C.I.	
				L.L	U.L.
HD^**^	-0.39	0.257	0.135	-0.91	0.12
RTR^***^	-0.004	0.233	0.985	-0.47	0.46
pH	-7.03	3.061	0.023	-13.18	-0.88
HD^**^	-0.31	0.279	0.273	-0.87	0.25
RTR^***^	0.06	0.235	0.793	-0.41	0.53
HCO3	-0.07	0.032	0.040	-0.13	-0.003
HD^**^	-0.37	0.253	0.144	-0.88	0.13
RTR^***^	0.04	0.228	0.857	-0.41	0.50
Acidosis	0.57	0.170	0.010	0.14	0.99

*Reference group: *CKD*: Chronic Kidney Disease, **HD: Hemodialysis

***RTR: Renal Transplant Recipients

Linear regression analysis of log UT II adjusted for LVM by group indicated that when switching from CDK to HD, a 55% decrease in the mean of UII scores is to be expected (Table 5). Finally, UII levels were positively correlated with HDL- cholesterol blood levels. This association however did not reach a statistical significance (*p* = 0.056). There was no correlation between UII and total cholesterol, LDL - cholesterol and triglycerides levels (*p* = 0.948, *p* = 0.882 and *p* = 0.230 respectively).

**Table 5:** Linear regression analysis of log-UT II adjusted for LVMI, LVM and Heart Rate by group*

Factor	Coefficient	S.D	*P*-value	95% C.I.	
				L.L	U.L.
HD^**^	-0.53	0.28	0.065	-1.09	0.33
RTR^***^	0.17	0.25	0.489	-0.32	0.67
LVMI	0.00025	0.0026	0.921	-0.0049	0.0054
HD^**^	-0.59	0.29	0.047	-1.18	-0.009
RTR^***^	0.15	0.25	0.536	-0.34	0.65
LVM	0.0039	0.006	0.554	-0.009	0.017
HD^**^	-0.46	0.27	0.093	-1.02	0.80
RTR^***^	0.12	0.25	0.642	-0.38	0.61
HR	-0.004	0.005	0.448	-0.015	0.006

*LVMI* Left Ventricular Mass Index, *LVM* Left Ventricular Mass, *HR* Heart Rate

*Reference group: *CKD*: Chronic Kidney Disease, **HD: Hemodialysis, ***RTR: Renal Transplant Recipients

## Discussion

The current study indicates that children and adolescents with CKD, especially patients who underwent renal transplantation and patients with no dialysed CKD, have significantly higher plasma urotensin II (UII) levels compared to healthy controls. Moreover, plasma UII concentration was significantly increased at the end of the HD session and was similar to that of RTR and CKD patients and higher than healthy controls. The only factor that seems to affect significantly plasma UII levels is the presence of metabolic acidosis. The effect of left ventricular mass (LVM) in UII levels was of marginal significance.

Plasma UII levels have hardly been studied in children and adolescents with renal diseases. A study in children with minimal change nephrotic syndrome revealed that plasma UII levels were lower and urinary UII levels were higher in relapse than those in remission. This difference was attributed to proteinuria. Moreover, patients, in relapse and in remission, had lower plasma and urine UII concentrations compared to healthy controls. Plasma UII had a strong correlation with plasma albumin, only in remission [22]. More dense UII immunoreactivity in renal biopsy specimens of children with membranoproliferative glomerulonephritis compared to healthy kidneys has been found and a possible autocrine/paracrine function of UII in kidneys has been considered [23].

To our knowledge there are no studies of plasma UII in children and adolescents with chronic kidney disease or in renal transplant recipients.

A few studies have evaluated plasma UII levels in adults with CKD or ESKD on HD and only one in RTR adults. The contradictory results of these studies may be mainly due to different methodology such as the heterogeneous populations of patients and controls, and the assays used for the determination of UII levels.

The first study of plasma UII-like immunoreactivity in adults with CKD was published fourteen years ago [9]. Plasma UII levels were higher in patients with CKD, especially those with ESKD on HD, compared to controls, although UII levels did not correlate with serum creatinine. Contrary to our study no significant change of UII levels at the end of the HD session was noted. Totsune et al assumed that increased UII may be the result of the decreased excretion from the kidney or of increased production. In addition, it was found that mRNA encoding UII precursor and its receptor was expressed not only in renal but also in many other tissues such as in the heart [9]. Three years later the same authors reported 1.6 times higher plasma UII levels in diabetic patients with severe CKD compared to patients with mild to moderate CKD. Furthermore, patients with more severe disease had 1.8 times higher urinary UII excretion than healthy controls [10].

Twofold higher plasma UII levels were found in patients with ESKD on hemodialysis (HD) compared to healthy controls in another study. Moreover, UII was an independent, inverse predictor of cardiovascular (CV) events in those patients [17]. An inverse correlation has also been observed between UII and some biomarkers of atherosclerosis and endothelial activation in ESKD patients undergoing HD. Furthermore, UII levels were lower in patients who received antihypertensive medication [28]. However, it remains unclear whether the hypertensive treatment or the hypertension influences UII levels [29]. A negative association between UII and CV stress hormones such as norepinephrine and brain natriuretic peptide (BNP) in patients on HD has also been considered, suggesting that higher UII concentration may be vasculoprotective [30]. The results of all the above mentioned studies contradict previous ones in which a positive correlation between increased UII and cardiovascular diseases was reported [31, 32]. A direct association of circulating UII with left ventricular systolic function and an inverse association with left atrial volume were considered, by Zoccali et al. [33], in the same patients recorded by Mallamaci F et al. [28]. These data further support the hypothesis that high UII is cardioprotective in adult ESKD patients.

Similarly, UII concentration was inversely correlated with the history of CV events as well with a lower risk of death from CV or other causes in adults with earlier stages of CKD [18].

Higher urinary UII-like immunoreactivity was found in hypertensive adults with normal renal function compared to controls. Furthermore, the hypertensive patients with renal disease had higher urinary UII-like immunoreactivity than normotensive patients with renal disease [34]. Greater plasma UII concentration in hypertensive compared to normotensive subjects has also been considered [6]. Increased expression of UII-related peptide and its receptor’s mRNAs in the kidneys of rats with hypertension and chronic kidney failure has been established [35].

Contrary to other studies, Mosenkis A et al., showed lower UII concentrations mainly in CKD as well in ESRD patients than controls. UII was negatively correlated with serum creatinine and the stage of CKD and positively with creatinine clearance. However the authors noticed an increase in UII levels at the end of the HD session [36]. Furthermore, in a recent study, predialysis of UII levels were lower in overhydrated compared to normohydrated ESKD patients [37]. Taking into consideration previous reports on the relationship between low UII concentration and the risk of cardiovascular events in patients with CKD, the authors assume that low levels may be a therapeutic target in overhydrated CKD patients [37].

It is speculated, that the removal of fluids from our patients by HD and the resulting hemoconcentration may have a role in the increase of HDII levels after the completion of HD [36, 37]. Moreover, UII is a middle size molecule (molecular mass = 1388.6 g/mol) and is only partially removed using the convential HD [36, 38]. In addition, the removal of fluids which leads to a reduction in blood pressure and to the improvement of many biochemical parameters, may have as a result an increased production of UII by the heart or other tissues [9, 36].

According to our results, there was no significant correlation found between UII and blood pressure measurements. These results agree with Mosenkis et al., who considered that this might be due to antihypertensive treatment [36].

Another recent study evaluated, for the first time, plasma UII levels in adults with RTR. The authors found significantly higher UII levels in RTR compared to CKD and to healthy controls.. This could not be explained solely by the transplanted kidney, because UII levels were higher than healthy controls and It was assumed that this could be immunosuppressive drug-related. However, the immunosuppressive drug doses and the duration of transplantation did not correlate with UII levels [39]. In our study, RTR children had significantly higher UII levels compared to healthy controls and higher but without statistical significance UII levels than CKD patients. There was also no correlation between UII and the duration of renal transplantation. We cannot support that immunosuppressive treatment was responsible for higher UII concentration of RRT patients, because of the small number of participants.

Another finding of our study was the correlation between UII and metabolic acidosis. The increase of pH was associated with a decrease of UII which was independent from all other parameters. There are no studies evaluating the correlation between these two markers. A study in human smooth muscle cells showed that UII affected intracellular pH inducing acidosis [40]. The high percentage of RTR patients with metabolic acidosis might be immunosuppressive drug-related [41]. A correlation of metabolic acidosis and protein catabolism is documented in a number of studies [42–44]. Protein catabolism might trigger the increase of UII.

UII levels did not differ significantly between males and females of this study and did not correlate with age in patients and controls. This is in accordance with most of the above mentioned studies [9, 36, 45]. On the contrary, a significant correlation between age and UII has been reported [6] and Hurshitoglu et al [39] observed that males tended to have higher levels of UII than females.

The main limitations of this study is the small number of participants per group and the absence of repeated measurements of UII after a certain period of time.

## Conclusions

Children and adolescents with chronic kidney disease, as well as renal transplant recipients, have significantly higher urotensin II levels than healthy controls. This could not be attributed solely to renal failure, since renal transplanted subjects had higher levels than healthy controls, non dialyzed CKD and HD patients. The effect of immunosuppressive therapy may play a role for these findings. The increase of urotensin II concentration at the end of the HD session might be the result of the correction of overhydration, the changes in biochemical and clinical parameters as well as the use of conventional hemodialysis.

Larger prospective studies of children with renal disease are required in order to establish plasma urotensin II levels and clarify its importance regarding the evaluation, follow up and management of these patients.

## Abbreviations

BMI: Body mass index
BP: Blood pressure
BSA: Body surface area
BW: Body weight
CKD: Chronic kidney disease
CV: Cardiovascular
DBP: Diastolic blood pressure
eGFR: estimated Glomerular Filtration Rate
ESKD: End-stage kidney disease
GM: Geometric mean
HD: Hemodialysis
HR: Heart rate
Ht: Height
LVM: Left ventricular mass
LVMI: Left ventricular mass index
OB: Obese
OW: Overweight
RTR: Renal transplant recipients
SBP: Systolic blood pressure
SD: Standard deviation
SDS: Standard deviation scores
UII: Urotensin II
